# Ensemble of Gene Signatures Identifies Novel Biomarkers in Colorectal Cancer Activated through PPARγ and TNFα Signaling

**DOI:** 10.1371/journal.pone.0072638

**Published:** 2013-08-19

**Authors:** Stefano Maria Pagnotta, Carmelo Laudanna, Massimo Pancione, Lina Sabatino, Carolina Votino, Andrea Remo, Luigi Cerulo, Pietro Zoppoli, Erminia Manfrin, Vittorio Colantuoni, Michele Ceccarelli

**Affiliations:** 1 Department of Sciences and Technologies, University of Sannio, Benevento, Italy; 2 Bioinformatics Laboratory, BIOGEM scrl, Ariano Irpino, Italy; 3 Department of Pathology, Hospital "Mater Salutis”, Legnano, Italy; 4 Institute for Cancer Genetics, Columbia University, New York, United States of America; 5 Department of Surgery and Oncology, University of Verona, Italy; The Chinese University of Hong Kong, Hong Kong

## Abstract

We describe a novel bioinformatic and translational pathology approach, gene Signature Finder Algorithm (gSFA) to identify biomarkers associated with Colorectal Cancer (CRC) survival. Here a robust set of CRC markers is selected by an ensemble method. By using a dataset of 232 gene expression profiles, gSFA discovers 16 highly significant small gene signatures. Analysis of dichotomies generated by the signatures results in a set of 133 samples stably classified in good prognosis group and 56 samples in poor prognosis group, whereas 43 remain unreliably classified. *AKAP12*, *DCBLD2*, *NT5E* and *SPON1* are particularly represented in the signatures and selected for validation *in vivo* on two independent patients cohorts comprising 140 tumor tissues and 60 matched normal tissues. Their expression and regulatory programs are investigated *in vitro*. We show that the coupled expression of *NT5E* and *DCBLD2* robustly stratifies our patients in two groups (one of which with 100% survival at five years). We show that *NT5E* is a target of the TNF-α signaling *in vitro*; the tumor suppressor PPARγ acts as a novel *NT5E* antagonist that positively and concomitantly regulates *DCBLD2* in a cancer cell context-dependent manner.

## Introduction

Colorectal Cancer (CRC) is one of the most common malignancies worldwide and a prevalent cause of morbidity and mortality. CRC survival is closely related to the clinical and pathological stage of the disease at diagnosis; over one third of CRC patients die within five years from the initial diagnosis and most of fatal outcomes result from liver metastases [1].

Despite the recent introduction of more effective therapeutic agents, there are only few validated prognostic biomarkers to assess the aggressiveness of the disease and the likelihood of recurrence or death after surgery. Recent studies propose small gene signatures as hallmarks of tumor stage [1,2]. Up to date integrative studies discovered amplifications of *ERBB2* and *IGF2* and genes significantly mutated in CRC such as *APC, TP53, SMAD4, PIK3CA* and *KRAS* as potential therapeutic targets [3]. Thus, the identification of accurate predictive and prognostic markers combined with the growing arsenal of therapeutic agents will provide more effective treatments related to the patient’s molecular profile minimizing life-threatening toxicities [4].

We developed a novel computational approach, gene Signature Finder Algorithm (gSFA) to generate several small gene sets which stratify the patients in terms of survival. Our strategy makes use of the availability of large-scale gene expression datasets to select candidates that can be then validated in independent libraries of tissues. We approached the problem of extracting suitable features from global gene expression that best correlate with the clinical information to create prognostic signatures. Most of the current procedures are based on expert knowledge to select, among thousands of genes, molecular markers that can be associated with prognosis [5]. Recently, novel methods, grounded on the data mining, machine learning [6] and statistical regression [7] for “Signature learning'' have been proposed. This is an interesting topic in Computational Biology and can be modeled as a problem of optimal feature selection [8]. Here, we adopted as optimality criterion the significance of the log-rank test between the survival curves of the groups induced by the selected features and used a novel procedure that integrates several signatures generated by a basic greedy algorithm. Signature genes are then ranked on the basis of some score metrics that measure the contribution of the gene to the signatures it belongs.

Starting from a public dataset of two hundred and thirty-two CRC gene expression profiles, our algorithm selected, among others, survival-related biomarkers such as *AKAP12, DCBLD2, NT5E*, and novel CRC-specific markers such as *SPON1*. We screened the candidate genes on two independent cohorts of 140 patients with primary sporadic CRC by using immunohistochemistry on Tissue MicroArrays (TMAs). The variations in gene expression were subsequently tested in an *in vitro* cell system that confirmed the *in vivo* data. Collectively, our data provide a new method to identify novel and robust biomarkers as a valuable step towards a better prognostic stratification and management of patients.

## Material and Methods

### Microarray Datasets

We apply *gSFA* (described below) to public datasets to identify a set of biomarkers. The data taken into account are those from the collections reported in [9] and available as GSE17536 and GSE17537 dataset in the Gene Expression Omnibus (GEO) (www.ncbi.nlm.nih.gov/geo). Both datasets are gene expression profiling obtained by using the Affymetrix GeneChip Human Genome U133 Plus 2.0 Array. GSE17536 counts 177 samples on 54613 gene-probes, while GSE17537 has 55 samples on the same probes. The 232 raw cell files were downloaded from both collections, then background correction, quantile normalization and summarization were applied.

### Tumor Samples

We analyzed CRC samples from two independent patients’ cohorts comprising a test series and a validation series (I and II), respectively. Cohort I comprises ninety-eight CRC cases and 60 paired apparently normal mucosa removed during the same surgery. This dataset includes both paraffin embedded and liquid nitrogen frozen specimens, as reported [10,11]. Cohort II consists of 42 cases of sporadic CRC collected at the Department of Pathology and Oncology, Legnago Hospital Verona, Italy during the period 2005-2007. Tumors were classified and graded according to the criteria of the TNM and tumor stages I-IV classification systems; the mean age of patients was 71.2±18.21. None of the patients had a familial history of intestinal dysfunction or CRC, had received chemotherapy or radiation prior to resection nor had taken non-steroidal anti-inflammatory drugs on a regular basis. Conventional post-operative treatments were provided to all patients, depending upon the severity of the disease. Overall length of survival was calculated starting from the first surgery. Patients were followed up for a median of 55 months or until death.

### Immunohistochemical analysis and evaluation of staining

For immunohistochemical analysis, tissue sections were deparaffinized, hydrated in graded alcohol, and microwave treated for 20 min at 750 W. Heat-induced epitope retrieval was performed by heating the TMA slides immersed in retrieval buffer citrate (pH 6.0). The sections were incubated with 3% hydrogen peroxide for 20 minutes at room temperature and subsequently with the specific antibodies: AKAP12 (dilution 1:500; WH0009590M1, mouse monoclonal, Sigma-Aldrich, St. Louis, MO), Spondin1 (dilution 1:250; AB40797; rabbit polyclonal, Abcam, Cambridge, UK) ); NT5E (dilution 1:50; AB115289; rabbit polyclonal, Abcam, Cambridge, UK); and DCBLD2 (dilution 1:100; AB115451; rabbit polyclonal, Abcam, Cambridge, UK) all antibodies were incubated overnight at 4°C. Secondary antibodies, followed by streptavidin-horseradish system were incubated for 30 minutes each, by using streptavidin-biotin peroxidase staining kit (LSAB+System- HRP; Dako Cytomation, Glostrup, Denmark). Immunoreactivity was revealed by incubation in 3,3-diaminobenzidine (DAB) substrate for 5 minutes. Subsequently, the sections were counterstained with hematoxylin, de-hydrated, and cover-slipped. Primary antibodies were omitted in negative controls.

A semiquantitative approach was used to evaluate immunoreactivity taking into account the number of positive cells and staining distribution of the staining in subcellular compartment (cytosolic and/or membrane). For each sample, the entire piece of micro tissue was examined through light microscopy at 20x magnification. The percentage of cancer cells, identified by immunoreactivity for each marker, was estimated for all samples in the TMA. Representative areas (5 high power field) containing the highest proportion of cancer cells were used for counting the tumor cells per section. The fraction (percentage of immunoreactive tumor cells, in triplicate samples) expressed as the number of Positive Cells for Fields (PCFs), was then calculated.

### Ethics Statement

This study was carried out according to the principles of the Declaration of Helsinki and approved by the Institutional Review Board of “FatebeneFratelli” Hospital in Benevento and Legnago Hospital, Verona, Italy. All patients provided written informed consent for the collection of samples and subsequent analysis.

### Statistical analysis: gene Signature Finder Algorithm (gSFA)

**Figure 1:**
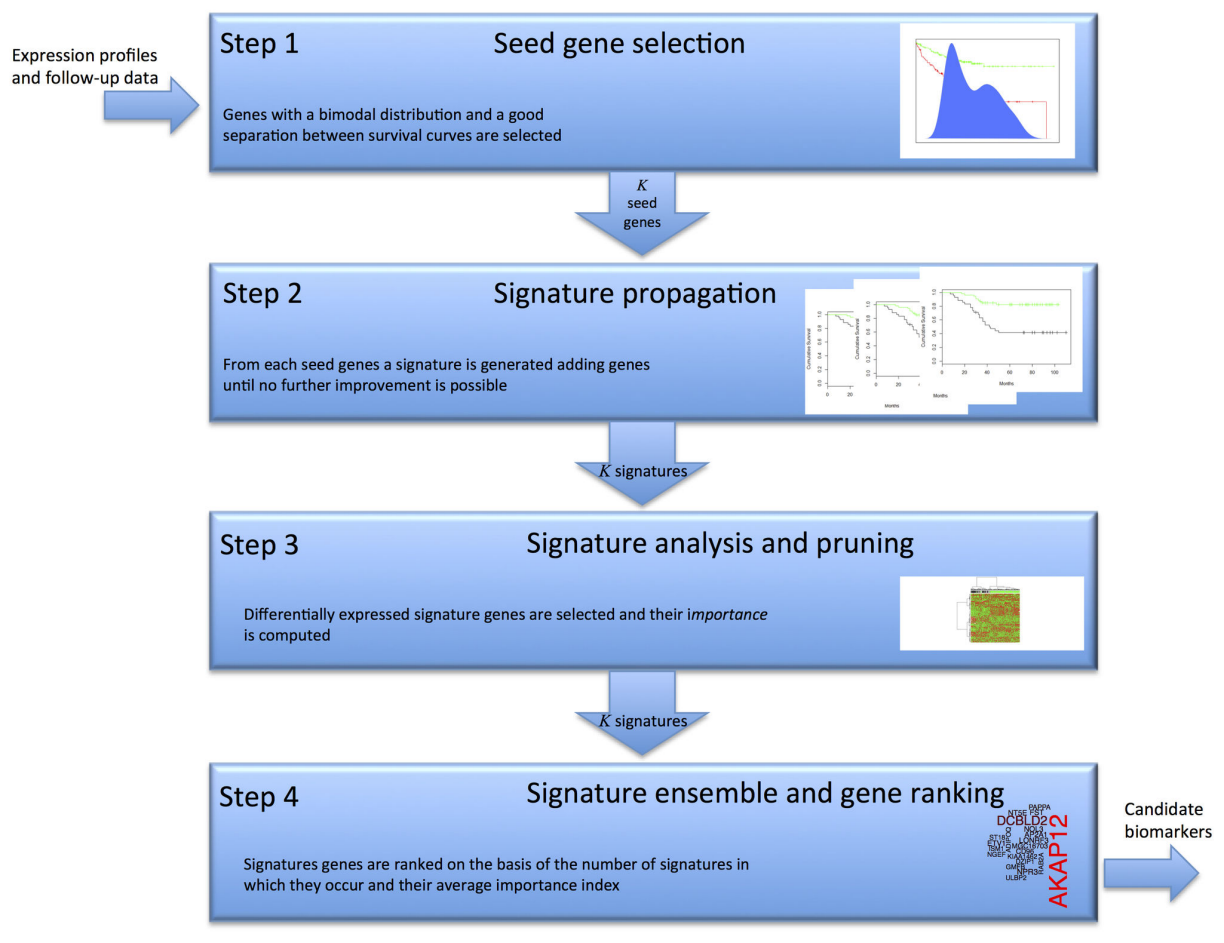
Outline of the gSFA procedure. The *gene Signature Finder Algorithm* consists of 4 separate steps: (1) find candidate seed genes; (2) generate a signature starting from each seed gene; (3) prune the signatures by statistical inference; (4) integrate signatures by gene ranking.

Our gene selection procedure employs an efficient wrapper method [8] based on unsupervised clustering, with a perturbation scheme generating several gene-sets with different initial conditions to start gene selection. The initial conditions correspond to seed genes that exhibit some properties such as being a good initial hypothesis. The resulting algorithm is defined *geneSignatureFinder* (gSFA) implemented in an R-package and available on CRAN (http://cran.r-project.org/web/packages/geneSignatureFinder/index.html) in an open source for download. gSFA, that is a generalization of the modified Steepest Descent proposed by Boutros et al. [6], consists of four main steps (Figure 1):

- Step 1: *Seeds Finder*
  The first step of our algorithm is aimed at selecting a set of genes that can be a reliable starting seed to expand the signature. We used a set of genes according to two main conditions: (a) a bimodal distribution of the expression levels, (b) ability to separate the dataset in two groups on the basis of the survival analysis test. In particular, for each gene *i* considers the sequence g_*i*_={*x*_*ij*_, *i*=1…, *M*}, where *x*_*ij*_ is the expression level of gene *i* in the sample *j*. The Bayesian Information Criterion (BIC) [12] is then used to check the bimodality hypothesis for the sequence g_*i*_. The sequence g_*i*_ is clustered in two subsets S_1_ and S_2_ by using a *k*-median algorithm (*k* = 2) and the distance between the survival curves of the samples in S_1_ and S_2_ is then computed. Seed genes are those manifesting bimodality and separation between the curves under a chosen significance threshold (in all our experiments 0.05).
- Step 2: *Signature propagation*
  Given a set of *K* seed genes selected as the previous step, we expanded a a small gene signature starting from each seed gene. A greedy strategy is adopted by selecting as next signature gene the gene maximizing the increase of the distance between the survival curves of the two sets S_1_ and S_2_ obtained by clustering the two-dimensional dataset (containing of the seed gene and the next candidate signature gene). The algorithm continue adding genes to each signature in this way until no further improvement of the distance between survival curves is obtained.
- Step 3: *Signature analysis and pruning*
  An *importance index* is computed for each gene of the *K* signatures, it is a measure of how much a single gene contributes to improve the distance between the survival curves with respect to the distance based on all genes in a signature. It is defined as 1 minus the ratio of the distances of the survival curves obtained when the selected gene is removed from the signature (details in the accompanying file Supplementary gSFA procedure). Then each signature is pruned by the genes that do not significantly contribute to the separation of the dichotomy generated by the signature.
- Step 4: *Gene Ranking*
  From the ensemble of gene in the *K* signatures, we can obtain various scores to select candidate genes that are potentially associated with the prognostic groups. We score the genes on the basis of the number of signatures in which they occur and their average importance index.

The accompanying Materials & Methods S1 (Supplementary gSFA procedure) contains all the details of the algorithm and the commands to replicate the whole analysis with R using the gSFA package.

## Results

### gene Signature Finder Algorithm (gSFA) reveals many signatures associated with cell adhesion and extracellular matrix organization

**Figure 2:**
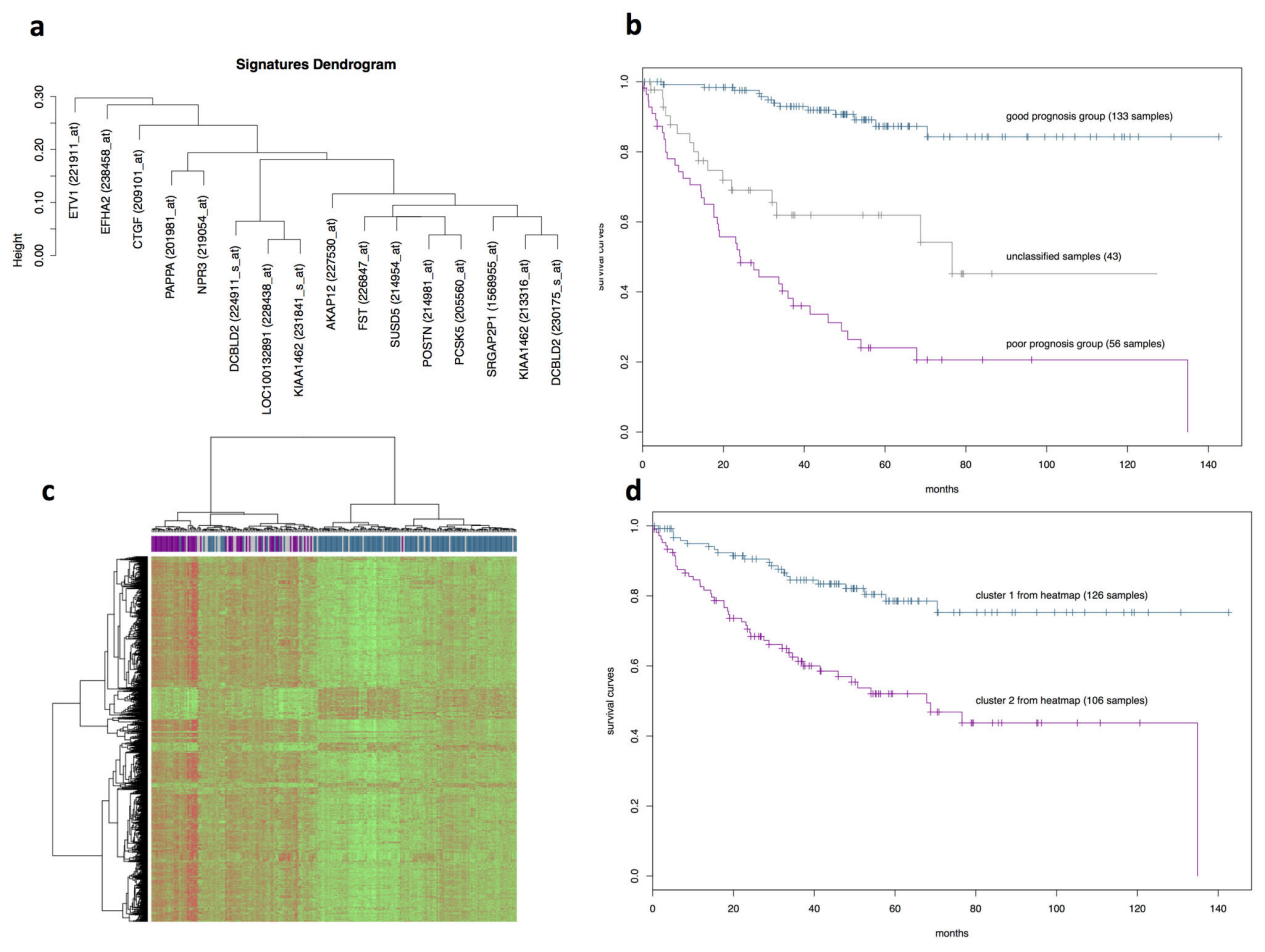
Analysis of the signatures generated by gSFA. **a**. Dendrogram of the dichotomies generated by each of the 16 gSFA signatures (Table 1 and Figure S1); **b**. Survival plot of the 133 samples stably classified in the good prognosis group, 56 samples in the poor prognosis group and 43 uncertain samples (log-rank test gives *p* < 10^--16^); **c**. heatmap of DEGs between the two stable groups, Red indicates overexpressed genes (expression levels over the median) and green indicates underexpressed genes (expression levels under the median); **d**. survival curves samples in the two clusters of the heatmap as in **c**. (*p* = 6.6 ·10^-6^).

We applied the gSFA to a dataset of two hundred and thirty-two CRC samples and identified 16 seed-genes that show a significant (*p* < 0.05 after correction) univariate separation of the survival curves between good and poor prognosis groups, and are bi-modal at the same time (about 1/3 of the probes show bimodality). From each of the seed-genes we obtained the signatures that are listed in Table 1; the corresponding survival curves are illustrated in Figure S1. In most cases, the *p*-value is not computable given that the *t*-value is beyond the machine precision. Although the selected gene subsets can be different, the survival curves often appear similar (Figure S1), so we asked the difference between the separation induced by each signature. We computed the distance between clustering obtained by each of the 16 signatures and get the dendrogram reported in Figure 2a: we observe that different gene signature can eventually generate similar dichotomies. This dendrogram allows to obtain a robust classification of the samples in two groups according to the number of times a given sample falls in one of the groups. In particular, the *stable good prognosis group* comprises samples that in at least 80% of dichotomies fall in the good prognosis group, analogously for the *stable poor prognosis group*. Thus, 133 and 56 samples were classified in the stable good or poor prognosis group, respectively, whereas the 43 remained unreliably classified and defined uncertain samples. The corresponding survival curves are reported in Figure 2b, log-rank test between the three curves gives a *p*-value < 10^-16^. 1609 differentially expressed genes (fold change 1.5 and corrected *p*-value < 0.05) between the two stable prognostic groups generated the clustering heatmap of Figure 2c. The samples were divided in two main clusters: the first comprised 113 out of 133 (85%) of the stably classified as good prognosis group; the second 55 out of 56 (98%) of the poor prognosis group. The corresponding survival curves are reported in Figure 2d, the log-rank test between the two curves gives *p* = 6.6·10^-6^. The silhouette of the cluster separation is also reported in the accompanying file Supplementary gSFA procedure.

**Table 1:** Signatures developed from 16 seed-genes.

**Seed Gene**	***t*-value**	**log(*p*-value)**	**Signature**
PCSK5 (205560_at)	76.572	<-16	PCSK5, AKAP12, NPR3, AGPAT5, GMFB, C6orf141, 1569202_x_at, KCNH8
FST (226847_at)	75.757	<-16	FST, AKAP12, ULBP2, SLC25A43, EI24, 1563467_at, CLDN8
POSTN (214981_at)	74.023	<-16	POSTN, AKAP12, AGPAT5, ATL3, SLC44A2
SUSD5 (214954_at)	71.842	<-16	AKAP12, 241867_at, ADAMTS5, APLP2, PITPNC1, 1556983_a_at
KIAA1462 (231841_s_at)	67.624	-15.654	KIAA1462, DCBLD2, ADIPOQ, FAM217B, C17orf48
DCBLD2 (230175_s_at)	66.71	-15.477	DCBLD2, AKAP12, GUSBP11, CDR2L, MGC16703, METTL4
NPR3 (219054_at)	66.457	-15.477	NPR3, DZIP1, 243820_at, 238109_at, 236795_at, DNAJC4, FOXA1, EMID2
AKAP12 (227530_at)	65.522	-15.256	AKAP12, ISM1, C11orf9, 244026_at, ARHGAP9, NOL3, AP2A1
PAPPA (201981_at)	65.024	-15.109	PAPPA, NT5E, DUSP7, 230711_at, CD96, ABI2
ETV1 (221911_at)	61.631	-14.386	ETV1, LONRF3, NGEF, RAB2A, U2AF2, CPO
KIAA1462 (213316_at)	60.733	-14.184	KIAA1462, AKAP12, UGGT2, 231989_s_at
SRGAP2P1 (1568955_at)	58.417	-13.674	SRGAP2P1, AKAP12, SNX16, NT5E
LOC100132891 (228438_at)	58.037	-13.589	LOC100132891, DCBLD2, ADIPOQ, SLFN5
DCBLD2 (224911_s_at)	50.106	-11.837	DCBLD2, ADCY7, EHD2
CTGF (209101_at)	46.099	-10.949	CTGF, FERMT1, AKAP12, CDK1
EFHA2 (238458_at)	42.166	-10.076	EFHA2, ST18, ACACB

To elucidate the biological processes and functions associated with the these two clusters, we identified 1394 differentially expressed probesets corresponding to 1024 different genes (DEGs), 87% of which are contained in the list of DEGs between the stable prognostic groups. The DEGs list enriched several Gene Ontology (GO) categories. Our gSFA procedure selected, as related to the poor prognosis group, a set of samples with mesenchymal features associated with by genes involved in proliferation and cell adhesion. In particular, GO analysis of genes up-regulated in the poor group showed that *Cell Adhesion* was enriched with 123 DEGs (*p* < 10^-28^), *Extracellular Matrix Organization* with 34 genes (*p* < 10^-15^), *Response to Wounding* with 95 DEGs (*p* < 10^-22^), *Immune response* with 91 genes (*p* < 10^-14^). Moreover, these DEGs enriched the KEGG Pathways *ECM-receptor interaction* (*p* < 10^-10^) and *Focal adhesion* (*p* < 10^-8^). When loaded in the Ingenuity Pathway Analysis, our list of DEGs produced a set of enriched categories whose *p*-value is reported in Figure S2a. In particular, other categories related to tissue development and cancer were significantly enriched; specifically those related to colorectal cancer were enriched at *p* < 10^-14^.

In order to understand the key regulators involved in the separation in the two prognostic groups, an enrichment analysis of upstream regulators was performed. Interestingly, transforming growth factor beta 1 (TGFβ1) was up-regulated in the poor prognosis cluster [13]. Moreover, the network of the 20 regulators depicted in Figure S2b could explain the behavior of 177 of the selected genes. The top regulators of the network also comprised other soluble growth factors such as members of the fibroblast growth factor (FGF) and tumor necrosis factor (TNF) families. The selected genes are involved mainly in the TGFβ1 signaling, as 109 molecules (*p* < 10^-51^) are its annotated targets in the IPA knowledge base. Receptor mediated signaling in response to these ligands triggers the activation of intracellular effector molecules, such as, the small GTPase family RAS, members of the SRC tyrosine-kinase family or transcription factors including, SMAD, RUNX2, STAT3, NFκB, p53 and β-catenin. These effectors have a central role in CRC progression and metastatic invasion promoting the formation of a tumor-associated microenvironment.

### TMA validation of the biomarkers identified by the ensemble signatures

**Figure 3:**
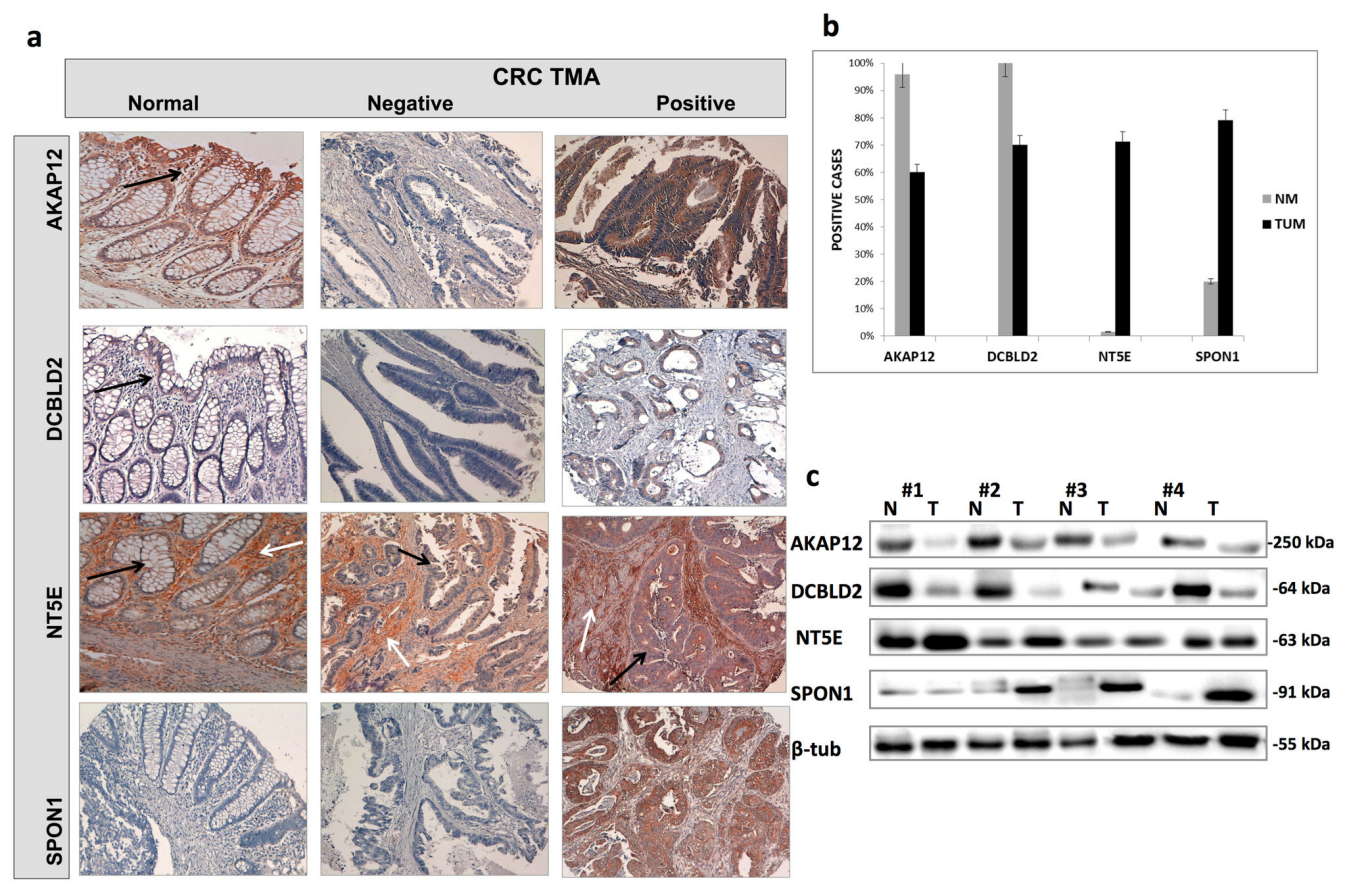
TMAs and western blot validation analysis of AKAP12, DCBLD2, NT5E and SPON1. **a**. “Columns from left to right” indicate immunostaining pattern in normal colonic samples and CRC cores negative and positive for each marker AKAP12, DCBLD2, NT5E, SPON1. Black arrows indicate immunohistochemical staining pattern in normal or malignant colonic cells. White arrows indicate the immunostaining distribution in the stromal compartment (endothelial, regulatory T-cells and macrophages) characteristic of NT5E expression pattern. Magnification 10X; **b**. Number of positive cases detected on TMA validation series comprising tumor specimens (TUM) and a subgroup of matched normal colonic mucosa (NM). Error bars indicate the standard deviation from the mean (*p* < 0.05); **c**. Four representative frozen CRC specimens (T) and matched normal mucosa (N) were identified in the same cohort of patients and analyzed by immunoblot. Molecular weight markers are indicated in kilodaltons. β-tubulin was used as loading control to normalize band intensities.

To identify robust biomarkers associated with CRC prognosis that can be validated in our two libraries of tissues, we used the ranking reported in the third step of gSFA and the resulting genes are shown in Table S1, where *AKAP12, DCBLD2, NT5E* were the most common genes selected by the algorithm in the various signatures. We also validated SPON1 as, unexpectedly, it was among the most differentially expressed genes of the two groups; however, its role in CRC has not been addressed yet. We screened by immunohistochemistry (IHC) the TMAs of our series of 140 CRCs and a subset of 60 normal matched colonic samples to determine the prognostic potential of the selected genes. Markers’ expression pattern was recorded as proportion of positive cells: a positive staining above 25% of tumor cells was defined high expression. Figure 3a reports representative tissue slides of the TMA labeled with antibodies against AKAP12, DCBLD2, NTE5 and SPON1. Figure 3b reports the overall percentages of detection between tumor and normal samples. In Figure S3 further staining of DCBLD2 and NT5E at a higher resolution is illustrated.

In the normal colonic mucosa, AKAP12 immunostaining was always positive, localized to the cytosol and mainly distributed in the apical crypt compartment i.e. in more differentiated cells. In CRC samples, AKAP12 immunoreactivity was retained in 55% and absent in about 45% of the cases.

DCBLD2 was always positive in normal colonic cells and evenly localized at the baso-lateral plasma membranes. In CRC samples, the immunostaining was detected in approximately 52% of the cases. In few tumor sections, DCBLD2 immunopositivity was also localized to the cytosolic compartment.

NT5E immunopositivity was mainly found in stromal cells (endothelial or regulatory T-cells) of the normal colonic mucosa, while it was weakly positive or negative in epithelial colonic cells. In line with this finding, NT5E immunopositivity marked the tumor-associated stroma and was absent in malignant cells in about 40% of tumor samples. Interestingly, in the remaining 60%, NT5E staining marked also malignant epithelial cells, in addition to the stromal ones.

SPON1 immunoreactivity was positive only in about 20% of normal colonic samples. Remarkably, SPON1 was detected in approximately 70% of CRC samples, with a membrane or cytosolic localization. Positivity within tumors was significantly correlated with collagen IV and vimentin expression, suggesting a role of SPON1 in the reorganization and remodeling of tumor extra cellular matrix (ECM) (data not shown).

### Western Blot Analysis On Primary CRC

To corroborate the IHC expression profile and to have more quantitative data, twenty randomly selected CRC specimens and matched normal mucosas from the same cohort of patients were analyzed by western blot, using the same antibodies employed in IHC. This analysis also verified the specificity of the antibodies. The bands obtained were quantitated by densitometry after normalization to β-tubulin for protein loading. AKAP12 and DCBLD2 were frequently detected at lower levels in tumor (T) than normal matched tissues (N). In contrast, NT5E and SPON1 expression showed significantly higher levels in tumors than controls. Figure 3c reports the bands corresponding to four representative samples, the quantization of which is reported in Figure S4a. Although the data referred only to 20 cases, they confirmed the specificity of the results and reinforced the differences between normal and tumor samples detected by IHC on TMAs.

### Biomarkers Expression Profiles and Clinico-pathological Parameters

We performed a multiple comparison test evaluating the IHC expression frequency of each biomarker and the clinico-pathological parameters. We found no association between AKAP12 or SPON1 gene expression profile and patients’ age, sex, tumor stage, differentiation or histology, presence of nodal and liver metastases. In contrast, loss of DCBLD2 was correlated with advanced stages of the disease (*p* = 0.021). In fact, 37.4% of the samples tested low for DCBLD2 expression was more frequently Stage IV-tumors as compared to 15% of the DCBLD2 high expressing ones. Accordingly, even cases associated with distant metastases had significantly lower levels of DCBLD2 expression than those without liver metastases (*p* = 5.71·10^-5^). Notably, also tumors expressing high levels of NT5E showed a similar susceptibility to metastatic tumors (*p* = 6.62·10^-4^). The association between biomarkers and the clinico-pathological parameters in our CRC dataset is illustrated in Table S2.

**Figure 4:**
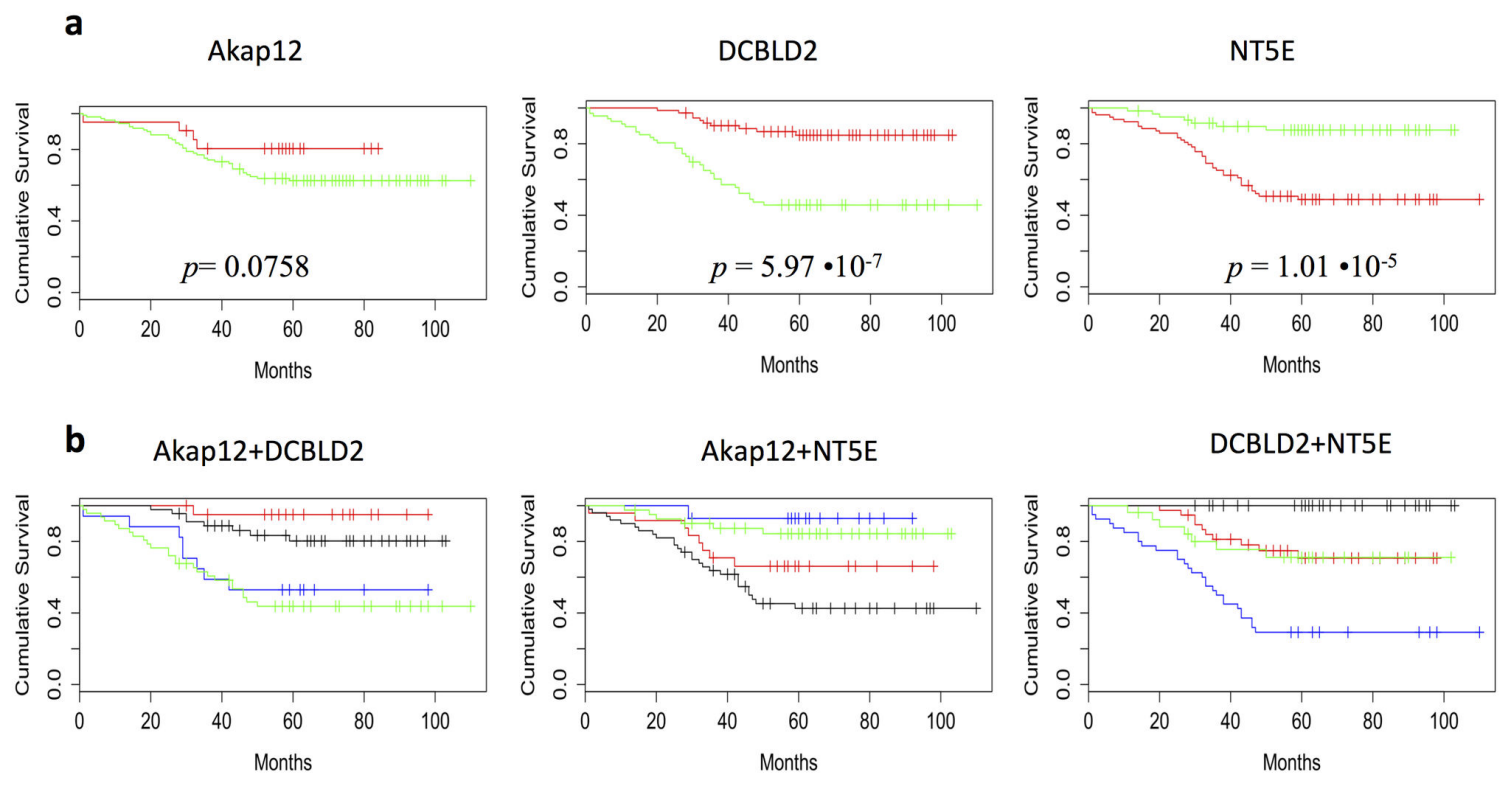
Survival curves of our cohort as function of the selected genes. **a**. Survival curves on our CRC validation series, categorized as having high (red curve) and low (green curve) AKAP12, DCBLD2 and NT5E expression. *p*-value for the null hypothesis of equal population survival curves is provided by log-rank test in each graph; **b**. Survival curves estimated combining the expression (*high* and *low*) of all 3 markers (AKAP12, DLBLC2, NTE5). Red curve represents the combination *high/high*; the blue curve represents the combination *high/low*; the black curve represents the combination *low/high*; the green curve represents the combination *low/low*.

To explore which of the selected genes was also an independent predictor of patient survival in our CRC validation series, we classified tumors as low or high expressing each of the biomarkers under investigation. Kaplan-Meier survival analysis revealed that loss of AKAP12 was marginally associated with poor overall survival (OS) (*p* = 0.0758, Figure 4a). Remarkably, low membrane-associated expression of DCBLD2 and overexpression of NT5E in tumor cells was strongly associated with shorter survival (*p*=5.97·10^-7^and *p*=1.01·10^-5^ respectively, Figure 4c and 4d). No significant association with OS was found taking into account only the NT5E immunopositivity in tumor associated-stroma (Figure S5). SPON1 over-expression was not correlated with patients’ prognosis (data not shown).

Finally, we evaluated whether all three predictive markers (AKAP12, DCBLD2, NT5E) could exert reciprocal effects and improve prognostic accuracy. The cases were classified into 4 groups according to their expression levels (Figure 4d, 4e, 4f). Notably, 100% of patients showing the combination DCBLD2^high^/NT5E^low^ were alive at 5 years after diagnosis. In contrast only 30% of patients whose tumors expressing the combination DCBLD2^low^/NT5E^high^ were alive. This survival difference was independent of adjuvant chemotherapy or stage of the disease.

### Expression of selected biomarkers in CRC cell lines

To gain further insights into the candidate genes, we assessed their protein expression profile in six representative human CRC derived cell lines (Figure S3b). AKAP12 protein was detected only in HCT116 and DLD1 cells according to previous studies [14]. DCBLD2 was expressed at very low levels in the majority of cell lines, while NT5E was highly expressed in HT29 and HCT116 as compared to the other cell lines. Finally SPON1 was detected in the majority of the cell lines, with the highest levels in DLD1.

### Cross-talk between TNF-α and PPARγ signaling regulates NT5E and DCBLD2

The *in vivo* results suggested that NT5E and DCBLD2 are involved in tumor invasion and metastasis likely through a tumor-induced immunosuppressive mechanism. Thus, we focused our attention on TNF-α, a cytokine that regulates a signaling network implicated in inflammatory diseases and tumorigenesis [15]. Ingenuity Pathway Analysis was queried to highlight the effects of TNF-α and interacting cytokines. NFκB emerged as the main hub of the network of upstream regulators of the selected DEGS and, in turn, as a crucial modulator of genes implicated in inflammatory and immune response (Figure S6a) [16]. Based on these observations, we searched for cis-acting regulatory DNA elements in *NT5E* and *DCBLD2* promoters (Figure S6b) and identified several putative NFκB site-like elements. To verify whether *NT5E*, and possibly *DCBLD2*, could be regulated by the inflammatory cascade, we treated HEK 293T cells with LPS, a powerful pro-inflammatory NFκB inducer, and obtained a significant time-dependent induction of *NT5E*; similarly, ectopic expression of p65/RelA NFκB subunit caused a significant induction further enhanced by the LPS treatment. In contrast, transfection of the super suppressor IκBα S32/36 fully abolished the LPS stimulatory effect on *NT5E* (Figures S6c and S6d). These results demonstrated that *NT5E* could be considered an NFκB target gene, expanding the list of known transcriptional activators such as β-catenin [17] and HIF-1α [18]. DCBLD2 was expressed at very low levels, if any, in HEK293T cells and no significant variations were appreciated following LPS treatment (data not shown). Albeit the limited sensitivity of the detection method, we cannot exclude that this gene is negatively regulated by the NFκB cascade.

**Figure 5:**
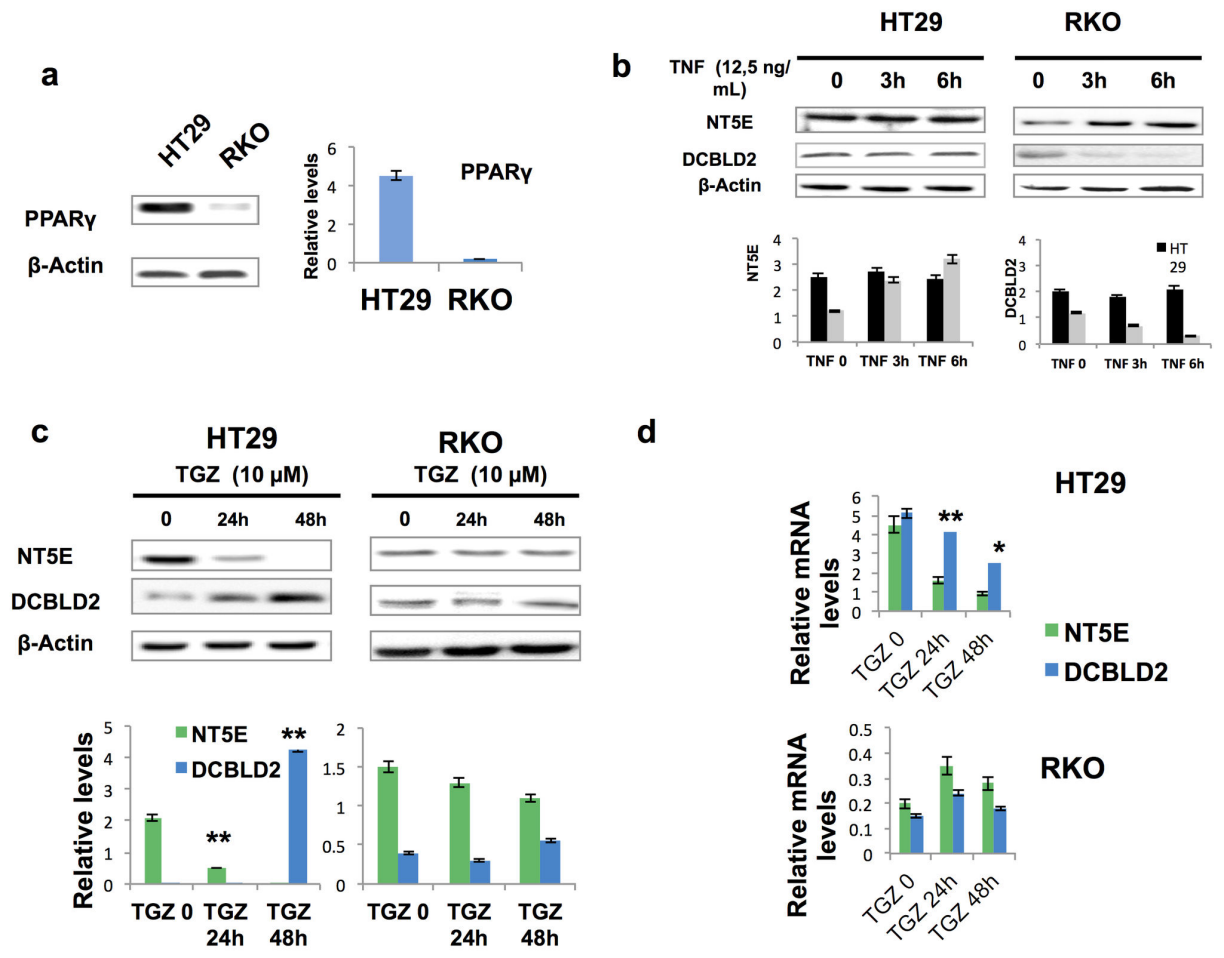
Cross-talk between TNF-α and PPARγ signaling regulates DCBLD2 and NT5E intracellular levels in CRC cell lines. **a**. Western blotting analysis shows PPARγ expression in HT29 and RKO CRC derived cell lines, as a model to investigate variations of NTE5 levels in response to TNF-α treatment. **b**. PPARγ-dependent protein induction of NT5E by TNF-α in CRC cells treated with 12,5 ng/ml TNF-α at different time points and analyzed by western blotting. **c** and **d**. Time course of *NT5E* and *DLBLC2* mRNA and protein regulation by troglitazone (TGZ), a PPARγ agonist, in the same cells. β-actin and Glyceraldehyde-3-phosphate dehydrogenase (GAPDH) were used as control to normalize expression levels in Western blotting and Real-time RT-PCR analysis. The results are expressed as means ±S.D. of three independent experiments. ^*^ *p* < 0.05, ^**^p < 0.01.

In the network of upstream regulators derived from the DEGs list, a role is played by PPARγ as a negative mediator of NFκB action (Figure S6a). This nuclear receptor is able, in fact, to restrain cell growth and preserve epithelial differentiation [10,19]. Since a peroxisome proliferator response element (PPRE) was present in the *NT5E* and *DCBLD2* proximal promoter, we hypothesized that both genes could respond to PPARγ signaling and their transcription modulated by a crosstalk with TNF-α signaling. To this goal, we used HT29 and RKO cell lines as representative of high or low PPARγ-expressing cells, respectively (Figures 5a and [10]). No significant effects on NT5E and DCBLD2 protein levels were detected in HT29 cells treated with TNF-α; conversely, a 2-fold induction of NT5E and a reduction of DCBLD2 was observed in RKO as compared to controls (Figure 5b). As expected, we did not detect any variation of *PPARG* in *RKO* following TNF-α treatment, as the gene is epigenetically silenced. In HT-29 cells we observed instead a 40% reduction of PPARγ protein but not of the corresponding mRNA as compared to controls (data not shown). These results indicate that PPARγ levels and/or activity can be affected by TNF-α signaling in a cell context-dependent manner, in keeping with literature data (15). We cannot exclude, however, that PPARγ protein–protein interactions or other “poorly understood” molecular mechanisms can modulate NT5E intracellular levels in response to TNF-α. To test the hypothesis that the results of the TNF-α treatment might depend upon PPARγ activity, we treated the cells with troglitazone (TGZ), a well-known PPARγ agonist able to induce apoptosis and block DNA synthesis [20,21]. Following exposure to TGZ (10 µM) for 48h, *NT5E* was dramatically down-regulated at the mRNA and protein level in HT29 cells and only slightly reduced at the protein level, with no changes of the mRNA in RKO cells (Figure 5c and 5d). In contrast, *DCBLD2*, that is barely expressed in both cell lines, showed a striking 4-fold increase only at the protein level in HT29 cells and no detectable variations in RKO cells (Figure 5c and 5d). GW9662, a specific PPARγ-inhibitor, completely blocked the effects reported on *NT5E* and *DCBLD2* in HT29 cells, confirming that they are PPARγ-dependent (data not shown). Altogether, these findings suggest that PPARγ expression/activation may directly affect the intracellular levels of NT5E and DCBLD2 by interfering with the TNF-α signaling.

## Discussion

Recent advances in high-throughput technologies allow unbiased genome wide screening of potential biomarkers or gene expression signatures that might predict prognosis [2,5–7]. Despite the significant progresses in understanding the molecular mechanisms underlying CRC progression, most therapeutic approaches are still based on clinico-pathological parameters. In the current study, we applied a new computational intensive gene selection procedure on genome-wide surveys of gene expression data to identify novel potential biomarker genes or a gene expression signature that could associate the biological and clinical characteristics of patients with prognosis. We applied a combination of signatures together with score metrics to measure the contribution of the gene to the signatures where it belongs. By using this novel approach, we identified and validated a number of genes whose expression patterns can predict patients’ survival. Gene expression data from two independent public dataset were used as a training set. The robustness of the signature as well as the genes selected was validated in our two CRC independent cohorts, comparing tumor samples with the matched normal mucosa. The algorithm was able to select novel survival-related biomarkers such as *AKAP12, DCBLD2, NT5E*, and several other differentially expressed genes. In our tumor validation set we focused on *AKAP12, DCBLD2, NT5E* with a higher stringent threshold cut-off, and *SPON1* whose expression appears to be highly cancer-related in the explorative “training series”. Tissue microarray technology was used to increase the throughput of this analysis. We tested the hypothesis that differences in the expression of these genes and related proteins could account for differences in the clinical outcome in our cohort of patients.

Protein kinase A anchor protein 12 (*AKAP12*) was one of the most representative genes of the signature that discriminates for patient’s prognosis in the public dataset analyzed. AKAP12 is a scaffold protein for PKA and PKC that regulates actin-cytoskeleton reorganization and inhibits SRC-mediated oncogenic signaling [22]. The gene is localized on chromosome 6q24-25.2, a deletion hotspot region in several cancers [23]. It has been implicated in tumor progression and indeed in our cohort its reduced expression correlates with a poor outcome, strengthening the hypothesis that it acts as a tumor suppressor in CRC [13,24]. *AKAP12* down-regulation is due to promoter methylation and a de-methylating agent such as 2’-5-Azacytidine, reactivates its transcription affecting downstream gene expression [25–27].

*SPON1/F-Spondin* is a member of the thrombospondin (TSR) gene family comprising trans-membrane proteins involved in regulating extracellular matrix organization, cell-cell interaction and axon guidance [28,29]. Recent studies suggest that alterations in “axon guidance” molecules are involved in several pathological processes [29–31]. *SPON1* contribution to CRC tumorigenesis is essentially unknown. We selected *SPON1* to prove its biological relevance in our TMA validation series. It robustly discriminated between tumor and normal samples but was not associated with patients’ survival. We hypothesize SPON1 as active mediator of tissue remodeling, promoting an aberrant cell-ECM interaction and acquisition of mesenchymal markers in malignant tissues (our data not shown) [31].

Discoidin, CUB and LCCL domain containing 2 (*DCBLD2*) was identified as one of the most enriched genes in the signature indicative of prognosis. *DCBLD2* was down-regulated in our CRC samples with respect to the normal adjacent mucosa and more frequently in patients with liver metastases and shorter overall survival. This gene belongs to a group of trans-membrane glycoproteins, known as neuropilins, with different biological roles as: a) they inhibit tumor angiogenesis and progression being specific antagonists of vascular endothelial growth factors; b) they act as receptors for axon guidance factors called semaphorins [32–35]. However, little is known about its involvement in CRC pathogenesis. In keeping with previous data, our results suggest that *DCBLD2* plays an important role in reducing tumor proliferation and metastasis in gastric cancer [33].

*NTE5/CD73* has recently received great attention through its ability to promote tumor immune surveillance evasion and metastasis [36]. NTE5/CD73 is a GPI-anchored cell surface enzyme expressed in hematopoietic and endothelial cells that converts AMP to adenosine, an immune suppressive molecule. It has also been identified as a component of 7-gene expression signature in stage III tumor patients, implying that it could serve as a prognostic and/or therapeutic target in CRC [2]. Despite a reinvigorated interest in *NT5E* expression, understanding of the precise role is still limited. In our CRC series, elevated levels of NT5E within the tumor microenvironment but also in malignant epithelial cells were strongly related to patients’ poor outcome [36,37].

We applied multiple pairwise combinations to verify whether reciprocal combinations of the selected genes could better predict patients’ clinical outcome. We identified NT5E^low^/DCBLD2^high^ and NT5E^high^/DCBLD2^low^ combinations that “robustly” differentiated the predictive and prognostic nature of the biomarkers. Remarkably, none of the patients presenting the NT5E^low^/DCBLD2^high^ combination died after surgery, whereas only 30% of patients with the NT5E^high^/DCBLD2^low^ combination were still alive 5 years from diagnosis.

Finally, we investigated the molecular mechanisms that regulate *NT5E* and *DCBLD2* expression. NFκB was identified as a potential central upstream regulator implicated in the TNF-α signaling that plays important roles in inflammation, tissue remodeling and cancer development. In vitro we validated *NT5E* as a novel NFκB target gene in addition to the hypoxic nature of the tumor microenvironment and the Wnt/β-catenin pathway, well established triggers of *NT5E* expression in tumor cells [17,18]. In this scenario, our previous results and present findings suggest that a variety of corrupted pathways in CRC could merge to activate *NT5E* expression and promote cancer growth and metastasis. In line with this, activation/over-expression of NFκB and β-catenin is frequently observed in aggressive CRCs [38,39]. Intriguingly, our algorithm identified PPARγ as a central player in the same regulatory network. This nuclear receptor is associated with a longer survival in our cohort of patients and interferes with the NFκB signaling in various cell contexts [19,38,39]. We hypothesized and confirmed *in vitro* that tumor cell sensitivity to TNF-α and the subsequent *NT5E* expression was PPARγ-dependent. Since NT5E gene response to TNF-α/NFκB appeared to inversely correlate with PPARγ, we hypothesize that also in CRC PPARγ counteracts TNF-α/NFκB activation, likely through a transrepression mechanism [40]. Even more interestingly, the PPARγ-dependent downregulation of *NT5E* was accompanied by *DCBLD2* upregulation. This positive modulation was observed only at protein level, suggesting that protein–protein interactions can modulate DCBLD2 intracellular levels, likely through still undefined mechanisms. Since *NT5E* and *DCLB2* inversely affect cell proliferation and associate with patients’ outcome, the interplay of TNF-α with PPARγ revealed by our approach could represent a pivotal relay-point in determining tumor progression towards a more aggressive behavior. The relevance of PPARγ and the interplay with TNF-α/NFκB as mediators of *NT5E* and *DCBLD2* functions requires further investigations at genome-wide level.

In conclusion, we developed a novel computational approach, *gene Signature Finder Algorithm* (gSFA) to generate several small gene-sets that stratify patients according to survival. By applying this procedure, we validated new prognostic biomarkers and found PPARγ and TNF-α signaling as novel regulators of NT5E and DCBLD2 levels. The gene signature reflects the molecular characteristics of the patients and provides an opportunity for the rational identification of novel biomarkers of potential clinical benefit. The method proposed, if implemented and confirmed in prospective studies, can open new therapeutic possibilities and effective guidelines in a variety of human malignancies.

## Supporting Information

Figure S1 **Survival curves for the 16 signatures of Table 1.** Each signature is labeled with the corresponding seed gene are reported in Table 1.
(JPEG)

Figure S2 **IPA Pathway Analysis of DEGs between cluster 1 and cluster 2.** a. Enriched Biological Functions as reported by IPA from the list of 1024 DEGs between cluster 1 and cluster 2; b. Regulatory interaction network showing the central role TGF-β1, FGF and TNF signaling pathways. This network was algorithmically generated mapping DEGs to known biological relationships. The network of 20 modulators controls 17% of DEGs. Regulator elements are represented as nodes and their interactions as edge.
(JPEG)

Figure S3 **DCBLD2 and NT5E specific staining in CRC.** **a**,**b**. CRC section (T) and adjacent normal mucosa (Nm) stained with DCBLD2 and NT5E. Black arrow indicates NT5E positive staining in stromal cells; negative NT5E staining in normal and malignant epithelial cells is observed. **c**,**d**. immunopositivity of DCBLD2 and NT5E in two CRC samples. DCBLD2 is abundantly expressed in membrane and cytosolic compartment. NT5E marks intensely malignant epithelial cells and stromal compartment (black arrow). Magnification 20X.
(JPEG)

Figure S4 **Biomarkers expression levels in tumor tissues and CRC derived cell lines.** a. Expression levels of each biomarker detected by western blot analysis in 4 representative paired tumor and normal tissues. The box-plots show expression profiles in a group of 20 representative tumor samples (T) and matched normal mucosa (N). The expression levels were normalized to that of β-tubulin by calculating the relative expression levels.
b. AKAP12, DCBLD2, NT5E and F-spondin levels were examined by western blot in the indicated CRC cell lines. The histogram reports their relative expression levels after normalization to b-tubulin.
(JPEG)

Figure S5 **Kaplan–Meier curve stratified on NT5E immunostaining in tumor associated stroma.** Red curve represents survival of samples with NT5E high expression in tumor stroma; the green curve represents survival of samples with low NT5E expression. The *p*-value was not significant *p* >0.05.
(JPEG)

Figure S6 **Signaling crosstalk between proinflammatory stimuli, NFκB pathways and NT5E and DCBLD2.** Interactive network of top 17 focus gene hubs centered on TNF signaling; NFκB is the most highly connected gene inhibited by PPARγ. **b**. site-like elements of NFκB and peroxisome proliferator response elements (PPRE) in *NT5E* and *DCBLD2* promoters. **c**. LPS stimulates NT5E expression in a time dependent manner in 293T cells. A vector encoding the super suppressor IκBα S32/36A (flag ss-Ikβa) abolishes the stimulatory effect of LPS on NT5E. d NFκB-p65-V expressing vector HA-tagged (HAp65V) determined a significant induction of NT5E, increased with LPS treatment. Expression is determined by western blot analysis. Bars represent mean values ±s.d. of three independent experiments relative to β-actin. **p<0.01.
(JPEG)

Table S1 **Gene ranking reports the most common genes selected by the algorithm in the various signatures.** The second column reports the number of signature containing the corresponding gene and third column is its average importance index.
(PDF)

Table S2 **Correlation of selected biomarker and clinico-pathological parameters** The classification of the tumors was based on the TNM (Tumor-Node-Metastasis) system according to the criteria of the International Union Against Cancer. In red are indicated statistically significant levels.
(PDF)

Materials & Methods S1 (PDF)
