# Supplementary figures and images for: Ensemble of Gene Signatures Identifies Novel Biomarkers in Colorectal Cancer Activated through PPARγ and TNFα Signaling
Source: PLoS One. 2013 Aug 19;8(8):e72638. doi: 10.1371/journal.pone.0072638 (PMC3795784; doi:10.1371/journal.pone.0072638)

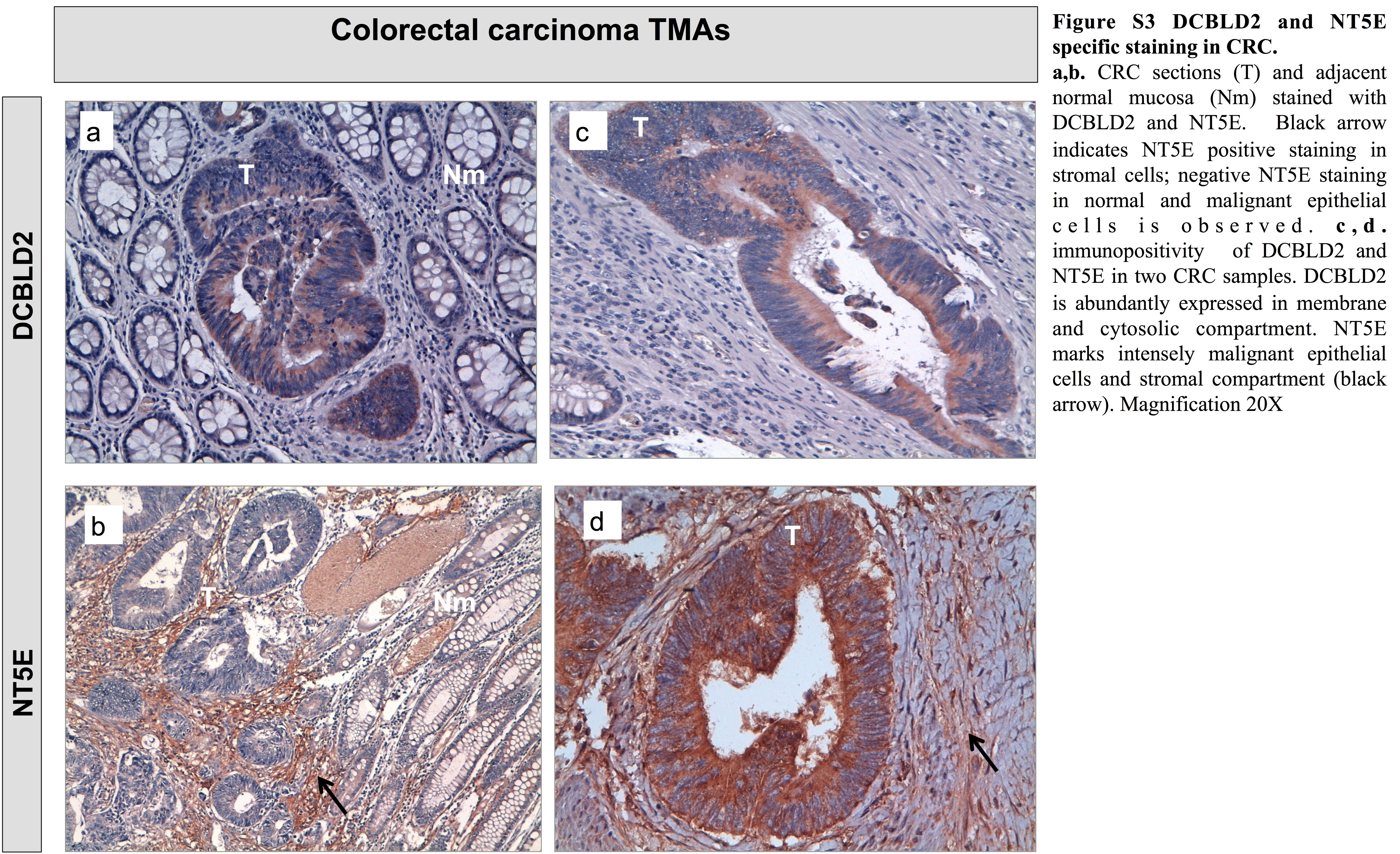

Supplement: Figure S3 — DCBLD2 and NT5E specific staining in CRC. a,b. CRC section (T) and adjacent normal mucosa (Nm) stained with DCBLD2 and NT5E. Black arrow indicates NT5E positive staining in stromal cells; negative NT5E staining in normal and malignant epithelial cells is observed. c,d. immunopositivity of DCBLD2 and NT5E in two CRC samples. DCBLD2 is abundantly expressed in membrane and cytosolic compartment. NT5E marks intensely malignant epithelial cells and stromal compartment (black arrow). Magnification 20X. (JPEG) [file pone.0072638.s003.jpg]

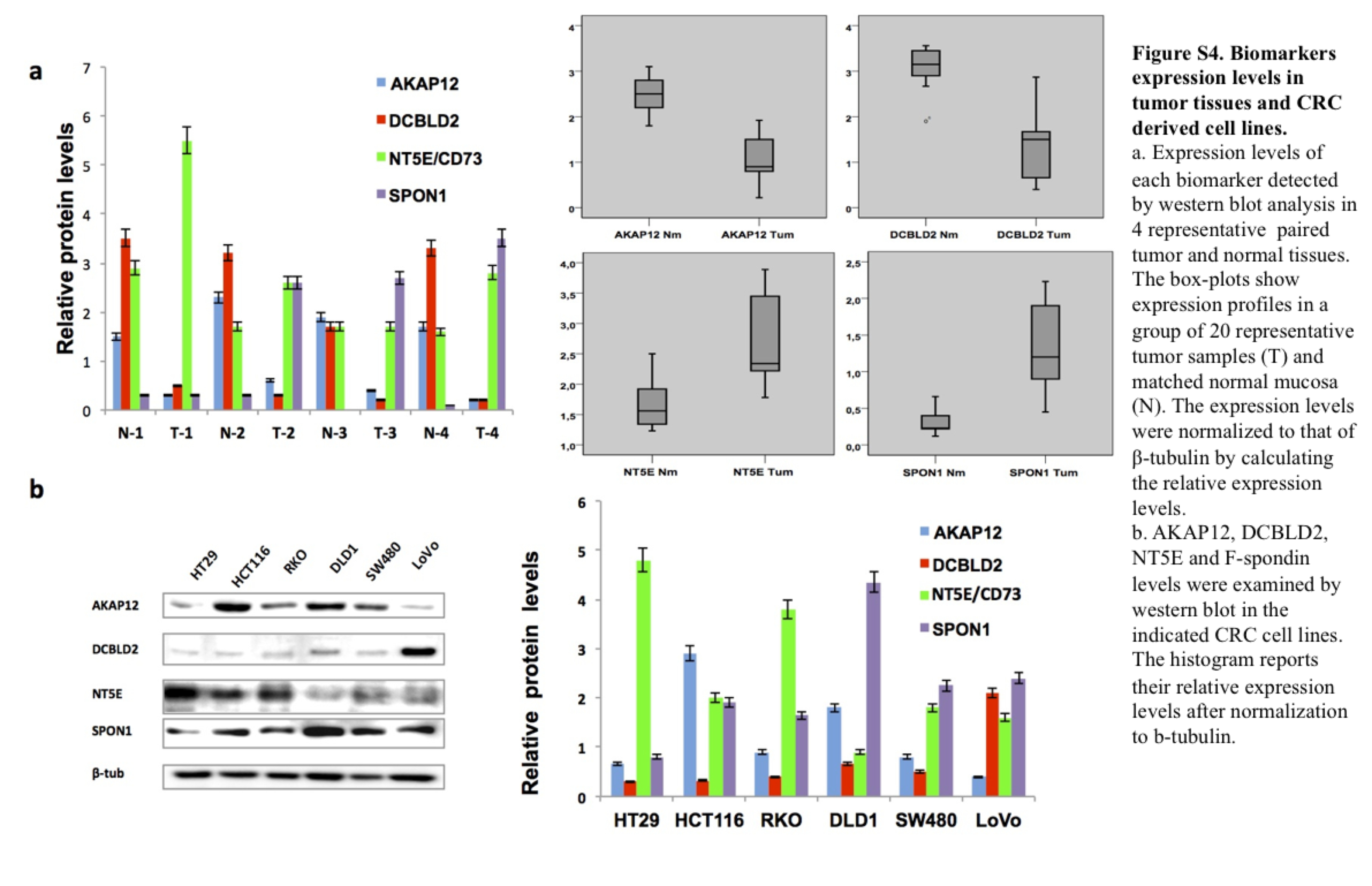

Supplement: Figure S4 — Biomarkers expression levels in tumor tissues and CRC derived cell lines. a. Expression levels of each biomarker detected by western blot analysis in 4 representative paired tumor and normal tissues. The box-plots show expression profiles in a group of 20 representative tumor samples (T) and matched normal mucosa (N). The expression levels were normalized to that of β-tubulin by calculating the relative expression levels. b. AKAP12, DCBLD2, NT5E and F-spondin levels were examined by western blot in the indicated CRC cell lines. The histogram reports their relative expression levels after normalization to b-tubulin. (JPEG) [file pone.0072638.s004.jpg]

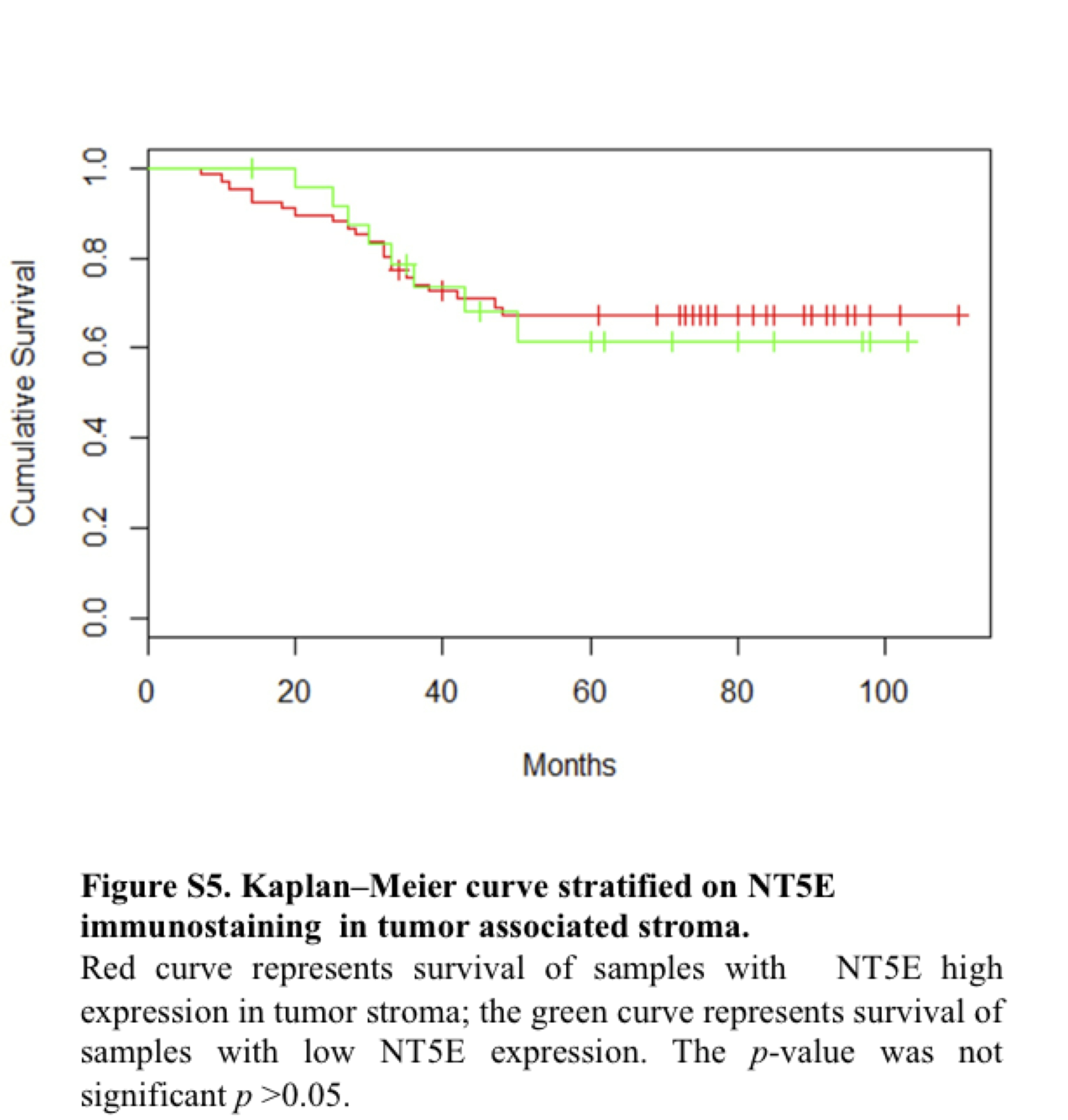

Supplement: Figure S5 — Kaplan–Meier curve stratified on NT5E immunostaining in tumor associated stroma. Red curve represents survival of samples with NT5E high expression in tumor stroma; the green curve represents survival of samples with low NT5E expression. The p-value was not significant p >0.05. (JPEG) [file pone.0072638.s005.jpg]
