# Supplementary material for: Ensemble of Gene Signatures Identifies Novel Biomarkers in Colorectal Cancer Activated through PPARγ and TNFα Signaling
Source: PLoS One. 2013 Aug 19;8(8):e72638. doi: 10.1371/journal.pone.0072638 (PMC3795784; doi:10.1371/journal.pone.0072638)
Supplement: Table S1 — Gene ranking reports the most common genes selected by the algorithm in the various signatures. The second column reports the number of signature containing the corresponding gene and third column is its average importance index. (PDF) [file pone.0072638.s007.pdf]

Table ST1. gene ranking reports the most common genes selected by the algorithm in the various signatures. The second column reports the number of signature containing the corresponding gene and third column is its average importance index.

Gene	Counts	Average importance
AKAP12	8	85.93
DCBLD2	3	79.31
NT5E	2	51.87
NPR3	2	54.74
AGPAT5	2	25.67
ADIPOQ	2	30.59
244026_at	1	54.46
SLFN5	1	27.81
243820_at	1	31.04
NGEF	1	69.11
LONRF3	1	92.05
241867_at	1	56.53
ULBP2	1	59.97
EFHA2	1	39.07
238109_at	1	35.5
FOXA1	1	17.26
236795_at	1	46.72
ISM1	1	71.58
METTL4	1	18.09
231989_s_at	1	12.7
KIAA1462	1	29.18
230711_at	1	35.93
DCBLD2	1	66.28
ADAMTS5	1	30.84
EMID2	1	10.99
LOC100132891	1	20.25
AKAP12	1	87.62
FST	1	99.37
FAM217B	1	33.84
SLC44A2	1	12.59
ARHGAP9	1	23.95
ATL3	1	35.78
AP2A1	1	95.31
ETV1	1	96.72
EHD2	1	11.03
NOL3	1	99.99
C17orf48	1	13.27

SNX16	1	35.38
PITPNC1	1	15.75
FERMT1	1	47.58
U2AF2	1	15.23
EI24	1	39.88
POSTN	1	40.43
CLDN8	1	19.16
DNAJC4	1	13.54
DUSP7	1	54.46
GUSBP11	1	29.28
KIAA1462	1	39.18
CDR2L	1	31.2
APLP2	1	24.59
CTGF	1	32.83
RAB2A	1	69.69
ABI2	1	30.36
ST18	1	63.36
PCSK5	1	36.88
DZIP1	1	57.28
C11orf9	1	50.99
ADCY7	1	30.69
CDK1	1	21.17
GMFB	1	56.85
PAPPA	1	84.23
1569202_x_at	1	42.03
SRGAP2P1	1	34.41
1563467_at	1	19.51
SLC25A43	1	23.38
1556983_a_at	1	48.01
UGGT2	1	45.31
CD96	1	81.97
CPO	1	12.64
KCNH8	1	44.47
MGC16703	1	86.56
ACACB	1	19.74
C6orf141	1	11.22
