# Supplementary material for: Ensemble of Gene Signatures Identifies Novel Biomarkers in Colorectal Cancer Activated through PPARγ and TNFα Signaling
Source: PLoS One. 2013 Aug 19;8(8):e72638. doi: 10.1371/journal.pone.0072638 (PMC3795784; doi:10.1371/journal.pone.0072638)
Supplement: Table S2 — Correlation of selected biomarker and clinico-pathological parameters The classification of the tumors was based on the TNM (Tumor-Node-Metastasis) system according to the criteria of the International Union Against Cancer. In red are indicated statistically significant levels. (PDF) [file pone.0072638.s008.pdf]

Table ST2. Correlation of selected biomarker and clinico-pathological parameters The classification of the tumors was based on the TNM (Tumor-Node-Metastasis) system according to the criteria of the International Union Against Cancer. In red are indicated statistically significant levels.

**Parameters**	AKAP12		*P*	DCBLD2		*P*	NT5E		*P*	SPON1		*P*
	Low %	High %		Low %	High %		Low %	High %		Low %	High %	
**Sex**			0.342			0.397			0.474			0.164
Male	56 (59.6%)	26 (68.4%)		45 (67.1%)	44 (60.2%)		40 (66.6%)	48 (60.75%)		21 (55.2%)	62 (68.1%)	
Female	38 (40.4%)	12 (31.6%)		22 (32.9%)	29 (39.8%)		20 (33.4%)	31 (39.25%)		17 (44.8%)	29 (31.9%)	
**Age**			0.376			0.265			0.222			0.197
≤60	16 (17%)	9 (23.6%)		15 (41.4%)	11 (15%)		14 (23.3%)	12 (15.2%)		10 (26.3%)	15 (16.5%)	
>60	78 (83%)	29 (86.4%)		52 (58.6%)	62 (85%)		46 (76.7%)	67 (84.8%)		28 (73.7%)	76 (83.5%)	
**Tumor Stage**			0.599			**0.021**			**0.005**			0.73
I	8 (8.5%)	6 (15.7%)		7 (10.4%)	8 (11%)		4 (6.6%)	11 (14%)		5 (13.1%)	9 (3.4%)	
II	43 (45.7%)	18 (52.9%)		26 (38.8%)	42 (57.5%)		37 (61.6%)	31 (39.2%)		20 (52.6%)	42 (55.9%)	
II	15 (15.9%)	5 (14.7%)		9 (13.4%)	12 (16.5%)		11 (18.4%)	9 (11.4%)		4 (10.5%)	15 (17%)	
IV	28 (29.9%)	9 (26.7%)		25 (37.4%)	11 (15%)		8 (13.4%)	28 (35.4%)		9 (23.8%)	25 (23.7%)	
**Grade**			0.262			0.682			**0.052**			0.726
G1	17 (18%)	10 (27%)		12 (17.9%)	15 (20.8%)		15 (25%)	12 (15.4%)		9 (24.3%)	18 (19.8%)	
G2	64 (68%)	25 (67.6%)		49 (73.1%)	48 (66.6%)		42 (70%)	54 (69.2%)		25 (67.6%)	62 (68.2%)	
G3	13 (14%)	2 (5.4%)		6 (9%)	9 (12.6%)		3 (5%)	12 (15.4%)		3 (8.1%)	11 (12%)	
**T Stage***			0.267			0.477			**0.012**			0.856
T1	5 (5.5%)	3 (8.6%)		2 (3.2%)	7 (10%)		5 (8.5%)	4 (5.5%)		2 (5%)	5 (5.7%)	
T2	8 (8.8%)	6 (17.1%)		7 (11.1%)	8 (11.4%)		2 (3.4%)	13 (17.8%)		5 (14.2%)	9 (10.3%)	
T3	72 (80.2%)	26 (74.3%)		52 (82.5%)	53 (75.7%)		52 (88.1%)	52 (71.2%)		26 (75.8%)	70 (80.4%)	
T4	5 (5.5%)	0		2 (3.2%)	2 (2.9%)		0	4 (5.5%)		2 (5%)	3 (3.6%)	
**N Stage**			0.466			0.095			0.488			0.513
N0	62 (65.95%)	27 (71.2%)		41 (61.2%)	43 (78.2%)		44 (73.8%)	52 (65.8%)		28 (73.7%)	60 (65.9%)	
N1	14 (14.90%)	7 (18.4%)		11 (16.4%)	7 (12.7%)		9 (14.2%)	12 (15.1%)		6 (15.8%)	14 (15.4%)	
N2	18 (19.15%)	4 (10.4%)		15 (22.4%)	5 (9.1%)		7 (12%)	15 (19.1%)		4 (10.5%)	17 (18.7%)	
**Distant metastasis**			0.453			**5.71E-05**			**6.62E-04**			0.612
M0	63 (67%)	28 (73.6%)		36 (53.7%)	62 (84.9%)		51 (85%)	46 (58.2%)		28 (73.7%)	63 (69.2%)	
M1	31 (33%)	10 (26.4%)		31 (46.3%)	11 (15.1%)		9 (15%)	33 (41.8%)		10 (26.3%)	28 (30.8%)	
