# Supplementary material for: Ensemble of Gene Signatures Identifies Novel Biomarkers in Colorectal Cancer Activated through PPARγ and TNFα Signaling
Source: PLoS One. 2013 Aug 19;8(8):e72638. doi: 10.1371/journal.pone.0072638 (PMC3795784; doi:10.1371/journal.pone.0072638)
Supplement: Materials & Methods S1 — (PDF) [file pone.0072638.s009.pdf]

Supplementary Information

Ensemble of Gene Signatures Reveals Novel Biomarkers in Colon Cancer through PPARg and TNF Signaling

Stefano Maria Pagnotta[1#], Carmelo Laudanna[1#], Massimo Pancione[1#], Lina Sabatino[1], Carolina Votino[1], Andrea Remo[3], Luigi Cerulo[1,2], Pietro Zoppoli[5], Erminia Manfrin[4], Vittorio Colantuoni[1*], Michele Ceccarelli[1,2*]

[1]Department of Science, University of Sannio, Benevento, Italy

[2]Bioinformatics Lab, BIOGEM scrl, Ariano Irpino, Italy

[3]Pathology Department, Hospital "Mater Salutis", Legnano, Italy

[4]Department of Surgery and Oncology, University of Verona, Italy

[5]Institute for Cancer Genetics, Columbia University, New York, USA

[#]Joint First Authors

[*]To whom correspondence should be addressed: michele.ceccarelli@unisannio.it, tel +39 (0)824305137 fax. +39 (0)824305142, vittorio.colantuoni@unisannio.it, tel. +39 (0)824305102, fax +39 (0)824305142

Material and Methods

Tissue MicroArray (TMA) Construction

TMAs were constructed from the tissue block using a Beecher tissue microarray instrument (Beecher Instruments, Hacken- sack, NJ, USA). Tissue cylinders, with a diameter of 0.6 mm, were punched from paraffin blocks of tumor or normal matched mucosa in demarcated areas on parallel haematoxylin and eosin-stained sections. Three separate foci of tumor and normal tissues were sampled from each block. The separate cores of tissue were deposited into a recipient master paraffin block. Each of the cores was placed 1 mm apart on the x-axis and 1.5 mm apart on the y-axis of the master block. In total cores of tumor and control mucosa were constructed in 8 microarrays. Sections 5μ thick were cut from the TMA master block and stained with haematoxylin and eosin. Microarray sections were then reviewed to ensure that the sections from each case were morphologically similar to those of the corresponding whole tissue section and represented cancerous or normal epithelial cells. Further sections 5 thick were then cut from each of the master blocks for immunohistochemical (IHC) analyses. The cores on TMAs containing too little tumor to allow an exhaustive analysis were not included in the study. Thus, the final numbers assessed were slightly variable for different markers, as a result of additional losses during block trimming and immunostaining procedures. Table S2 summarizes the patients' clinic-pathological characteristics in relation to each marker investigated.

Cell lines and culture conditions

In this study we used six human colorectal cancer cell lines (HT29, HCT116, RKO, DLD1, SW480, LoVo) obtained from the American Type Culture Collection (ATCC).
The cells were cultured in growth medium DMEM or RPMI-1640, supplemented with 10% fetal bovine serum (FBS), 100 U/μL penicillin, 100 μg/μL streptomycin and 2X L-glutamine at 37°C and 5% CO2.

DNA Constructs, Reagents, and Transfections

Immortalized, non-tumorigenic human HEK293T cells were cultured in DMEM supplemented with 10% FBS and maintained in a 5% CO2-humidified incubator. Cells were treated with a single dose (2 ug/ul) of lipopolysaccharide (LPS) (Cayman Chemical), a strong NFκB activator for 12 and 18 hours, respectively. HEK293T cells were transiently transfected with an expression vector coding for the super suppressor IKa mutated at Serines 32 and 36 (SS-IKBα), a competitor of the endogenous IKB molecules to block NFκB activation, or an empty vector as control [1]. Alternatively, the expression vector carrying the HA-tagged-p65 cDNA or the empty plasmid were transfected into cells exposed or not to LPS (2 ug/ul) for 18 hours [2]. All transfections were carried out with Lipofectamine 2000 (Invitrogen Corporation), according to the manufacturer's protocol and normalized transfection efficiency by CMV-LacZ. After the treatments, cells were harvested for protein extraction and immunoblot analysis as described below. TNF-α and Troglitazone (TGZ) reagents were purchased from (Roche Indianapolis, IN) and (Sigma Aldrich), respectively.
CRC cells (HT29 and RKO) were seeded in 24-well or 6-well plates and, once reached 70% confluence, were treated or not with a single dose of TNF-α (12,5 ng/ml) for 3 and 6 hours, or 10 uM TGZ for 24 and 48 hours respectively. The TGZ was reconstituted in Dimethylsulfoxide (DMSO) and control cells were incubated with an equivalent volume of DMSO vehicle only. Cells were harvested for RNA or protein extraction.

Western Blot analysis

Western blot analysis on protein extracts from CRC cell lines, tumor tissues and adjacent normal mucosa was performed using the procedure previously reported.
Cell lysates were prepared by resuspending cell pellets in lysis buffer (HEPES 50mM, pH 7.5, 150 mMNaCl, Glicerol 1%, Triton X100 1%, 1,5 mM MgCl2, 5mM EGTA 0.1M), supplemented with a mixture of protease inhibitors (Roche). After incubating on ice for 20 minutes, supernatants were isolated by centrifugation at 13,000 rpm for 20 minutes.
The following antibodies were used: anti-AKAP12; anti-DCBLD2; anti-NT5E; anti-SPON1; anti-HA (SC805) and anti-PPARγ (E-8) (Santa Cruz Biotechnology, Inc. Santa Cruz, CA, USA).

RNA extraction and Real-Time RT-PCR analysis

For the expression analysis, of *AKAP12*, *DCBLD2*, *NT5E* and *SPON1*, total RNA was isolated with Trizol (Invitrogen). Concentration of the total RNA was determined using the spectrophotometer (Genova Plus, Jenway). Reverse transcription-PCR (RT–PCR) was made using Superscript II (Invitrogen), 1ug of total RNA and random hexamer primers (Invitrogen). Quantitative RT-PCR was performed on 7300 Real-Time PCR System (Applied Biosystems, Foster City, CA, USA) using SYBR Green PCR Master Mix (Invitrogen),with 10 ng of cDNA as template and the specific primers as follows [3,4,5,6]:

- AKAP12: forward primer 5’-GGAGCCCTAAACAGCCAGGA-3’, reverse primer 5’-CTCCTCCTGCCCATCATCTG-3’, 150 bp
- DCLB2: 5’-CTCAGCCACTGGTAGGAGGA-3’m reverse primer 5’-GGCACCTGGTACACCAATTC-3’, 394 bp
- CD73 (NT5E): forward primer 5’-ATTGCAAAGTGGTTCAAAGTCA-3’, reverse primer 5’-ACACTTGGCCAGTAAAATAGGG-3’, 123 bp
- SPON1: forward primer 5’- CTCTTCCTGCAGAGGAGTAGTGTCA -3’, reverse primer 5’- CTGGGACTCAGGCATAGTCACTTC-3’, 90 bp.

Cycling conditions were as follows: 2 minutes at 50°C, 2 minutes at 95°C, 15 seconds at 95°C, annealing temperature of 60 secondsfollowed by 40 cycles with a final of dissociates stage. In all PCR reactions the genes of interest were normalized against the housekeeping gene glyceralaldehyde-3-phosphate dehydrogenase (GAPDH) RNA.

1. Bentires-Alj M, Barbu V, Fillet M, Chariot A, Relic B, Jacobs N, et al. NF-kappaB transcription factor induces drug resistance through MDR1 expression in cancer cells. Oncogene 2003; 22(1):90-7
2. Sun SC, Elwood J, Béraud C, Greene WC. Human T-cell leukemia virus type I Tax activation of NF-kappa B/Rel involves phosphorylation and degradation of I kappa B alpha and RelA (p65)-mediated induction of the c-rel gene. Mol Cell Biol. 1994; 14(11):7377-84.
3. Synnestvedt K, Furuta GT, Comerford KM, Louis N, Karhausen J, Eltzschig HK, et al. Ecto-5'-nucleotidase (CD73) regulation by hypoxia-inducible factor-1 mediates permeability changes in intestinal epithelia. Journal Clin. Invest. 2002;110(7):993-1002.
4. Hayashi M, Nomoto S, Kanda M, Okamura Y, Nishikawa Y, Yamada S, et al. Identification of the a kinase anchor protein 12 (*AKAP12*) gene as a candidate tumor suppressor of hepatocellular carcinoma. J. Surg. Oncol. 2012; 105:381–386.
5. Kim M, Lee KT, Jang HR, Kim JH, Noh SM, Song KS, et al. Epigenetic Down-Regulation and Suppressive Role of DCBLD2 in Gastric Cancer Cell Proliferation and Invasion. Mol Cancer Res. 2008; 6:222-230.
6. Shimada K, Uzawa K, Kato M, Endo Y, Shiiba M, Bukawa H, et al. Aberrant expression of RAB1A in human tongue cancer. Br. J. Cancer. 2005; 92(10):1915–1921.
